# Leo Kanner: The Physician and Pioneer of Autism

**DOI:** 10.7759/cureus.73859

**Published:** 2024-11-17

**Authors:** Angela Hamidi, Nadiya A Persaud, Latha Ganti, Leila Keeler

**Affiliations:** 1 Research, Orlando College of Osteopathic Medicine, Winter Garden, USA; 2 Emergency Medicine & Neurology, University of Central Florida, Orlando, USA; 3 Medical Science, Warren Alpert Medical School of Brown University, Providence, USA; 4 Specialty Care, Orlando College of Osteopathic Medicine, Winter Garden, USA

**Keywords:** autism, biography, clinical psychology and psychiatry, historical vignette, leo kanner, medical pioneer, psychiatry, psychology

## Abstract

Dr. Chaskel Leib (Leo) Kanner is widely regarded as the father of child psychiatry in the United States. He was a pioneering figure in autism research who paved the way for child psychiatry and our modern-day understanding of autism. His monumental efforts distinguished autism as a distinct neurodevelopmental condition, contributing to the establishment of specific diagnostic criteria that helped shape our current perception of autism. Kanner’s observations and theories recognized autism as an inherent condition, and his lasting contributions continue to influence diagnostic approaches while propelling ongoing, cutting-edge research.

## Introduction and background

Journey to advancement: childhood, education, and early career

**Figure 1:**
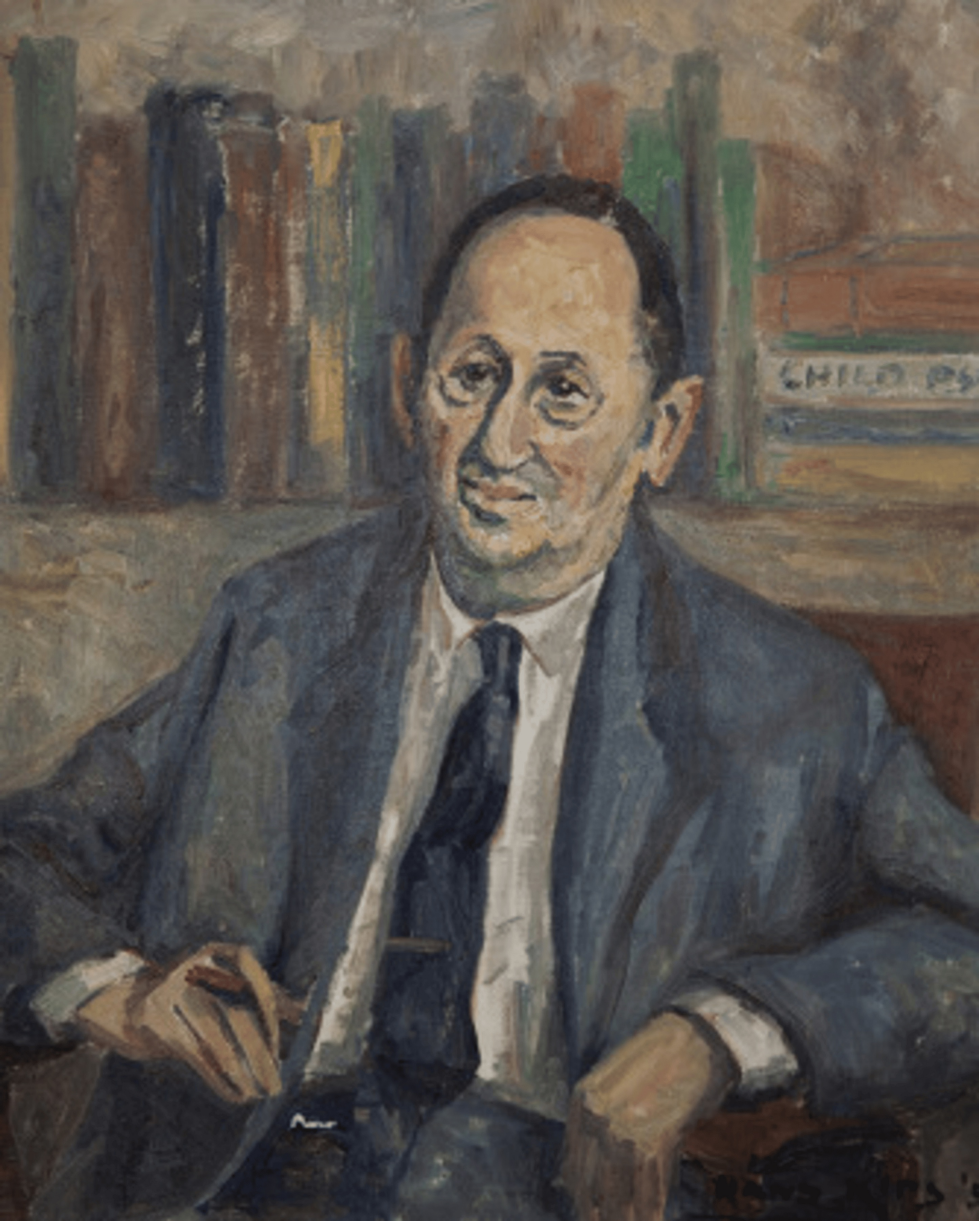
Dr. Chaskel Leib (Leo) Kanner Reproduced with permission from Lancet via Elsevier RightsLink [2]

Dr. Leo Kanner (Figure 1) was born in Austria in 1894 to Clara Reisfeld Kanner and Abraham Kanner [1]. At the ripe age of 12, he relocated to Berlin to further his education [2]. At the onset of World War I, Kanner dedicatedly served in the medical corps of the Austro-Hungarian army, an experience that greatly ignited his interest in medicine and provided invaluable early exposure to the field. Following the war, Kanner completed his medical education at the University of Berlin in 1921 and then joined Charité Hospital, one of Berlin’s leading medical institutions at the time [1], where he immersed himself in cardiology, focusing specifically on the study of normal heart sounds and their patterns on EKGs [2].

In 1924, following his marriage and the birth of his first child, Kanner made a transformative career move by emigrating to the United States to accept a position at Yankton State Hospital in South Dakota [2]. This transition became instrumental to his lasting contributions to medicine and psychiatry, shaping his future work in autism research. His writings at Yankton attracted the attention of prominent figures in psychiatry, including Adolf Meyer, a leading American psychiatrist of the era [2]. This recognition cemented Kanner’s reputation and profoundly shaped the trajectory of his pioneering contributions to the field of medicine and psychiatry.

In 1928, Kanner left Yankton to join Meyer at the Henry Phipps Clinic at Johns Hopkins University, where he would later go on to establish the nation’s first academic department of child psychiatry in 1930 [2]. This marked the beginning of Kanner’s lasting influence on child psychiatry and relentless advocacy for children, laying the groundwork for our modern understanding of autism. Kanner’s legacy endures in medicine and psychiatry, with his contributions resonating as we further today’s research and education in autism, solidifying his title as the father of child psychiatry in the United States [3]. This review aims to explore Dr. Leo Kanner's lifelong legacy and contributions to medicine, child psychiatry, and autism research, which continue to influence modern-day practices and diagnostic models.

## Review

The paper that set the stage

Kanner's 1943 academic paper, Autistic Disturbances of Affective Contact, set the stage for autism research by identifying key behaviors, patterns, and characteristics, leading to the establishment of initial diagnostic criteria. This pivotal work positioned autism on the map as a distinct neurodevelopmental condition [4]. The groundwork for this influential paper was laid in 1938 when he began observing 11 children at the Johns Hopkins clinic who exhibited behavioral traits he would later define as "early infantile autism," also known as Kanner's Syndrome [5-7]. In 1944, Kanner initially described early infantile autism as a form of schizophrenia that begins in infancy and continues throughout childhood, setting it apart from prior diagnoses such as mental deficiency, deaf-mutism, aphasia, and brain damage [8]. In the 1980s, the DSM-III (Diagnostic and Statistical Manual of Mental Disorders, Third Edition) classified infantile autism as its own distinct category. Asperger’s syndrome was later recognized in individuals displaying similar social and behavioral challenges, although absent of language delays and intellectual disabilities. This recognition contributed to the understanding that autism could manifest in a variety of ways, ultimately leading to the idea of autism as a spectrum and the adoption of the term “autism spectrum disorder” (ASD) [6]. Today, ASD is classified as a neurodevelopmental condition characterized by difficulties in social communication and interaction, often accompanied by restricted and repetitive behaviors, interests, or activities, echoing Kanner’s initial observations [9]. The term "spectrum" acknowledges the wide range of symptoms and severity levels unique to each individual’s experience of the disorder [9].

Over the course of the next five years, Kanner meticulously recorded the unique language patterns exhibited by his 11 participants (eight boys and three girls). He observed that while some children demonstrated a remarkable ability to memorize and recite phrases verbatim, they also encountered considerable difficulty in using language naturally and intuitively during social interactions. He also examined their intense infatuation and focus on specific objects and routines, suggesting that these behaviors were not merely responses to environmental stimuli but rather expressions of their underlying neurological framework [6].

Early misconceptions in Kanner’s findings and refrigerator mother theory

Although Kanner’s insights were foundational in understanding autism, several misconceptions emerged from his early findings. His use of the term “autism” drew parallels to characteristics of schizophrenia established by Dr. Eugen Bleuler, who associated self-centered thinking with the disorder. Although Kanner sought to distinguish autism from other psychiatric conditions, this association led some to assume that autism might be an early onset form of schizophrenia [4,10].

While Kanner recognized autism as a neurodevelopmental disorder, he initially attributed its causation to emotionally distant and absent parenting - a perspective that would later be known as the “refrigerator mother theory.” Bruno Bettelheim, a renowned U.S. psychologist at the time, popularized this theory, analogizing these cold, distant relationships to that of a refrigerator, emphasizing a perceived lack of warmth and detachment in parent-child relationships [4]. Although Dr. Bernard Rimland later disproved this theory, emphasizing that autism was not a consequence of parenting styles but rather of genetic and neurological factors, Kanner’s early research on inheritance patterns was instrumental in establishing the genetic and neurological foundations of autism [4,11]. In 1969, Kanner delivered a speech at a meeting of the National Society for Autistic Children, where he withdrew his earlier theory that emotional refrigeration caused autism, instead asserting that autism was an inherited condition [4,6]. Kanner also went on to publish his influential book, In Defense of Mothers: How to Bring up Children in Spite of the Zealous Psychologists, in which he offered reassurance to mothers, encouraging them to trust their own maternal instincts [6,11].

Role of genetic inheritance in the etiology of autism

After dissociating autism from parental nurturing, Kanner began advocating for a biological understanding of the condition in the early 1950s. The detailed accounts of his 11 participants highlighted a familial inheritance pattern, leading him to recognize genetic predispositions as influential in autism’s development. Kanner observed specific traits recurring in families, such as pronounced scientific, artistic, and literary interests, further supporting a genetic link to autism [4,6]. The recognition of these traits became instrumental in later studies measuring their prevalence in families through brain imaging, language assessments, and questionnaires, all of which align with Kanner’s early insights [6]. These observations foreshadowed what is now known as the broader autism phenotype, recognizing that specific autism-related traits may appear in family members who do not meet the full diagnostic criteria [4,6]. Kanner also observed an increased head circumference in some children with autism, a finding that echoes in modern research, showing that certain children with autism may experience rapid brain growth within the first 18 months of life [6,12]. Kanner’s observations were crucial in defining the role of genetic inheritance in the etiology of autism, establishing it as an inherent neurodevelopmental condition distinct from other psychiatric disorders and unrelated to parenting influences. His insights planted the seed for modern advancements in research and the development of diagnostic criteria.

Kanner’s influences can be seen in the current DSM-V model, which defines social communication deficits and restricted, repetitive behaviors as core diagnostic criteria. These criteria reflect Kanner’s early observations of autistic aloneness and insistence on sameness [6,12]. Kanner’s contributions greatly influenced our modern perspectives on autism including establishing diagnostic criteria and enhancing our understanding of the genetic traits associated with ASD.

Influence on other child psychiatrists

Dr. Leo Kanner’s early work served as an inspiration for Dr. Lorna Wing, a British psychiatrist. Wing expanded upon Kanner’s initial ideas, leading to a broader understanding of autism as a spectrum [7]. In a 1979 study with Judith Gould, Wing studied autism’s prevalence among children with special needs [7]. While their findings revealed rates of autism similar to those Kanner described in children with IQs below 70, they also identified a larger group exhibiting related challenges in social interaction, communication, and repetitive behaviors. Although these children did not meet Kanner’s full criteria for autism, Wing described them as being on a broader autism spectrum, introducing the concept of autism as a wide range of related conditions rather than a single syndrome [13]. This broader classification has since been validated by subsequent studies [14] and has greatly shaped our current understanding and diagnostic criteria for ASD.

## Conclusions

Dr. Leo Kanner’s abiding legacy continues to profoundly impact modern medicine and autism research. His influence is evident in the present-day diagnostic criteria for autism, having set the impetus for ongoing research and exploration into the condition. Kanner’s innovative research and publications were the first to define autism as a distinct condition rather than symptomatic of another disorder. Although he initially linked autism to the lack of parental nurturing, he later recanted this theory, recognizing autism as an innate condition. Kanner’s pioneering efforts in the 20th century have driven significant modern advancements in our understanding of autism and in strategies to best support those affected by it.
